# Replenishment of microRNA-188-5p restores the synaptic and cognitive deficits in 5XFAD Mouse Model of Alzheimer’s Disease

**DOI:** 10.1038/srep34433

**Published:** 2016-10-06

**Authors:** Kihwan Lee, Hyunju Kim, Kyongman An, Oh-Bin Kwon, Sungjun Park, Jin Hee Cha, Myoung-Hwan Kim, Yoontae Lee, Joung-Hun Kim, Kwangwook Cho, Hye-Sun Kim

**Affiliations:** 1 Department of Pharmacology, College of Medicine, Seoul National University, Seoul, 110-799, Republic of Korea; 2 Department of Life Science, POSTECH, Pohang, Gyeongbuk, 790-784, Republic of Korea; 3 Department of Physiology, College of Medicine, Seoul National University, Seoul, 110-799, Republic of Korea; 4 Henry Wellcome Laboratories for Integrative Neuroscience and Endocrinology (LINE), Faculty of Medicine and Dentistry, University of Bristol, Whitson Street, Bristol BS1 3NY, UK; 5 Centre for Synaptic Plasticity, School of Clinical Sciences, Faculty of Medicine and Dentistry, University of Bristol, Whitson Street, Bristol BS1 3NY, UK; 6 Seoul National University Bundang Hospital, College of Medicine, Seoul National University, Seongnam, Gyeonggi, 463-707, Republic of Korea

## Abstract

MicroRNAs have emerged as key factors in development, neurogenesis and synaptic functions in the central nervous system. In the present study, we investigated a pathophysiological significance of microRNA-188-5p (miR-188-5p) in Alzheimer’s disease (AD). We found that oligomeric Aβ_1-42_ treatment diminished miR-188-5p expression in primary hippocampal neuron cultures and that miR-188-5p rescued the Aβ_1-42_-mediated synapse elimination and synaptic dysfunctions. Moreover, the impairments in cognitive function and synaptic transmission observed in 7-month-old five familial AD (5XFAD) transgenic mice, were ameliorated via viral-mediated expression of miR-188-5p. miR-188-5p expression was down-regulated in the brain tissues from AD patients and 5XFAD mice. The addition of miR-188-5p rescued the reduction in dendritic spine density in the primary hippocampal neurons treated with oligomeric Aβ_1-42_ and cultured from 5XFAD mice. The reduction in the frequency of mEPSCs was also restored by addition of miR-188-5p. The impairments in basal fEPSPs and cognition observed in 7-month-old 5XFAD mice were ameliorated via the viral-mediated expression of miR-188-5p in the hippocampus. Furthermore, we found that miR-188 expression is CREB-dependent. Taken together, our results suggest that dysregulation of miR-188-5p expression contributes to the pathogenesis of AD by inducing synaptic dysfunction and cognitive deficits associated with Aβ-mediated pathophysiology in the disease.

MicroRNAs are non-coding RNA molecules with a length of approximately 22 nucleotides, which serve as post-transcriptional regulators of gene expression^1^,^2^. In the central nervous system, microRNAs have been shown to regulate development, survival, function and plasticity^3^,^4^,^5^.

MicroRNAs and their precursors exist in synaptic fractions along with components of the microRNA machinery^6^, where they are poised to regulate neurotransmission. Furthermore, dysfunction of microRNAs within neurons and alterations in microRNA expression have been associated with the pathogenesis of neurodegenerative diseases such as Alzheimer’s disease (AD)^7^,^8^. However, little is known regarding whether restoring or reversal of deregulated microRNAs is capable of counteracting deficits in cognitive or synaptic dysfunctions in AD.

Since AD-mediated cognitive deficits have been postulated as synaptic by origin^9^,^10^, one area that has been extensively researched is the study of aberrant amyloid beta peptide_1-42_ (Aβ_1-42_)-mediated modulation of synaptic transmission and plasticity^11^. The most extensively documented synaptic phenomenon in this regard is long-term potentiation (LTP), which is inhibited by overexpression of APP genes^12^ and Aβ administration^13^. Previously, we reported that miR-188-5p is up-regulated by LTP induction^14^. The protein level of neuropilin-2 (Nrp-2), which was confirmed to be a direct target of miR-188-5p by performing a luciferase activity assay in our previous study^14^, was decreased during LTP induction. It is of interest whether atypical miR-188-5p expression can be seen in AD and leads to aberrant long-term synaptic plasticity, an underlying cellular mechanism of learning and memory^15^.

Nrp-2 has been previously reported to serve as a negative regulator of spine development and synaptic structure, together with its ligand, semaphorin-3F (Sema-3F)^16^. Nrps are 130- to 140-kDa single transmembrane spanning glycoproteins that function as receptors for class 3 semaphorins, polypeptides essential for axonal guidance^17^,^18^ and for members of the vascular endothelial growth factor (VEGF) family, angiogenic cytokines^16^,^18^,^19^,^20^. Nrp-1 serves as a receptor for Sema-3A, which induces the collapse of the neuronal growth cone^21^,^22^.

In this study, we found that oligomeric Aβ_1-42_ treatment diminished miR-188-5p expression in primary hippocampal neurons and that miR-188-5p rescued the Aβ_1-42_-mediated synapse elimination and synaptic dysfunctions. Moreover, the impairments in cognitive function and synaptic transmission observed in 7-month-old 5XFAD transgenic mice, which harbor 3 familial AD mutations of APP 695, namely the Swedish, Florida, and London mutations and 2 presenilin1 (PSEN1) mutations (M146L and L286V), were ameliorated via viral-mediated expression of miR-188-5p. miR-188-5p expression was reduced and Nrp-2 was up-regulated in brain tissues from AD patients and 5XFAD mice. miR-188 gene has a cAMP response element (CRE) in its potential promoter region which would be shared with chloride channel 5 (*Clcn 5*). We found that CREB regulates the transcription of miR-188. Taken together, our results indicate that the reduction in miR-188-5p, which is expressed in a synaptic activity-dependent manner, in the brains of AD patients may contribute to the defective synapse elimination and cognition observed in the disease.

## Results

### miR-188-5p was reduced in brain tissues from AD patients

**Figure 1:**
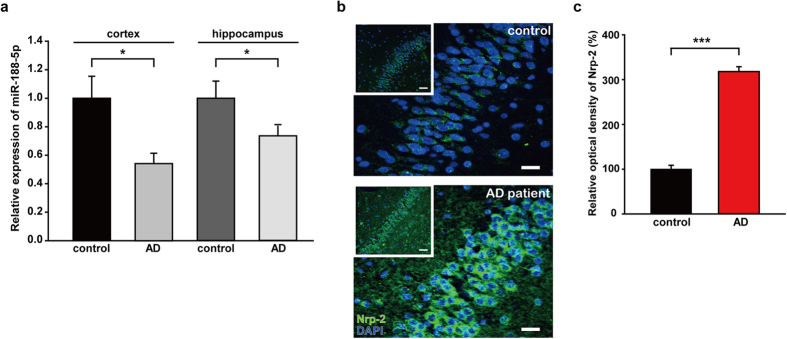
miR-188-5p was significantly down-regulated in the brains from AD patients. (**a**) miR-188-5p expression was examined by RT-qPCR in brains from AD patients and age-matched control subjects. miR-188-5p expression was significantly down-regulated in cerebral cortices (n = 3, vs. age-matched control subjects, n = 4, Mann-Whitney test) and hippocampi (n = 6, vs. age-matched control subjects, n = 4, Student’s t-test) of AD patients. (**b**) Representative images of the dentate gyrus of AD patients (98-year-old) compared with an age-matched control subjects. Nrp-2 immunoreactivity was measured by immunohistochemistry. Scale bars, 50 μm (inset, white square box) and 20 μm (magnified panel). (**c**) Quantitative graphs for Nrp-2 immunoreactivity in age-matched control subjects and AD (n = 3, Mann-Whitney test) and hippocampi (n = 4, Student’s *t*-test). Data are represented as the mean ± SEM. *p < 0.05, ***p < 0.001 compared to age-matched control subjects.

We examined miR-188-5p expression in the brain tissues of AD patients and age-matched control subjects by employing real-time quantitative PCR (RT-qPCR). Detailed information on the age-matched control subjects and AD patients used in this study is shown in Table 1. miR-188-5p expression was significantly down-regulated in the cerebral cortices (0.54 ± 0.07, *p* = 0.013) and hippocampi (0.74 ± 0.05, *p* = 0.038) of AD patients (Fig. 1a). Moreover, the immunoreactivity against Nrp-2, one of the molecular targets for miR-188-5p, was markedly increased (318.02 ± 10.86%, *p* < 0.001; Fig. 1b,c) in the hippocampi of AD patients compared with age-matched control subjects. However, *Nrp-2* mRNA expression was not significantly different from age-matched control subjects (Supplementary Fig. S1).

### Oligomeric Aβ_1-42_ reduced the expression of miR-188-5p

**Figure 2:**
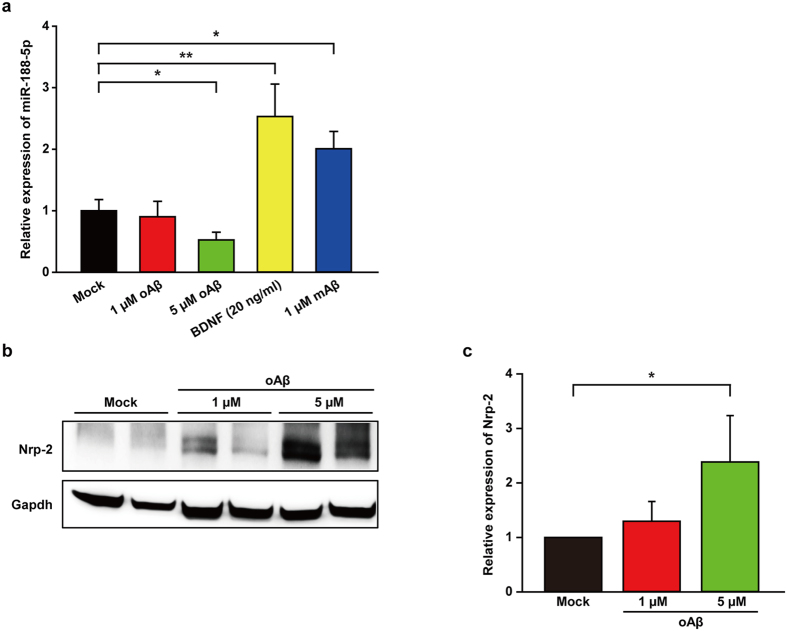
Oligomeric Aβ_1-42_ reduced the expression of miR-188-5p. (**a**) miR-188-5p expression was examined by RT-qPCR after treatment with oAβ in primary hippocampal neuron cultures. miR-188-5p expression was significantly reduced by treatment with 5 μM oAβ (n = 11, Mann-Whitney test) compared to vehicle-treated controls. (**b**) The Nrp-2 protein level was determined in primary hippocampal neuron after oAβ treatment by western blot (1 μM oAβ treatment and 5 μM oAβ treatment for 24 h compared with the control). (**c**) Quantitative graphs show relative quantification of Nrp-2 protein level normalized to Gapdh (internal control) after 24 h with 1 μM oAβ treatment (n = 3) or 5 μM oAβ treatment (n = 3, one-way ANOVA), compared to control (Mock). Data are represented as the mean ± SEM. *p < 0.05 compared to Mock. oAβ = oligomeric amyloid beta peptide_1-42_, mAβ = monomeric amyloid beta peptide_1-42_.

Aβ, which is the main component of neuritic plaques in AD brains, is thought to be a causative factor in the pathogenesis of the disease^23^. Among several aggregated forms of Aβ observed in AD brains, oligomeric Aβ has been reported to play the most important role in disconnecting the synaptic network^24^,^25^. We examined the effects of oligomeric Aβ_1-42_ on miR-188-5p expression and the protein level of Nrp-2, the molecular target of miR-188-5p in rat primary hippocampal neurons. Treatment with 5 μM oligomeric Aβ_1-42_ for 24 h significantly decreased miR-188-5p expression (0.52 ± 0.13 vs. vehicle-treated group, p = 0.03, n = 11, Fig. 2a), but increased Nrp-2 protein in the neurons (1 μM oligomeric Aβ_1-42_ for 24 h, 1.30 ± 0.36, *p* > 0.05 vs. vehicle-treated group; 5 μM oligomeric Aβ_1-42_ for 24 h, 2.38 ± 0.85, p = 0.037 vs. vehicle-treated group, Fig. 2b,c). Here, we confirmed that the treatment with 5 μM oligomeric Aβ_1-42_ for 24 h showed no significant difference in LDH release compared with vehicle treatment using a LDH assay (data not shown). Interestingly, we found that monomeric Aβ_1-42_ significantly increased miR-188-5p (2.01 ± 0.28 vs. vehicle-treated group, *p* = 0.02) in the neurons, which was not consistent with our expectations. Further research remains to determine the pathophysiological significance of this result.

We determined whether brain-derived neurotrophic factor (BDNF) affected miR-188-5p expression in the neuron cultures. BDNF is a neurotrophic factor that plays a pivotal role in synaptic plasticity and cognition^26^,^27^. Recently, it has been suggested that a decrease in BDNF within the prefrontal cortex and hippocampus is related to cognitive deficits in AD animal models^23^,^28^. Treatment with BDNF (20 ng/ml) significantly up-regulated miR-188-5p expression (2.53 ± 0.53 vs. vehicle treated group, *p* = 0.03; Fig. 2a).

### miR-188-5p rescued the Aβ-mediated reduction in dendritic spine density and basal synaptic transmission

Aggregation of oligomeric Aβ is thought to be a key pathophysiology^23^,^29^ and has been reported to play the most important role in neurotoxicity and neurodegeneration in AD^24^,^25^. Dendritic spine and synapse loss are well documented in AD^30^. It has been reported that Aβ decreases dendritic spine density in primary neurons^31^. In addition, the decrease in dendritic spine density was observed in the brains of AD animal model such as 5XFAD^32^.

**Figure 3:**
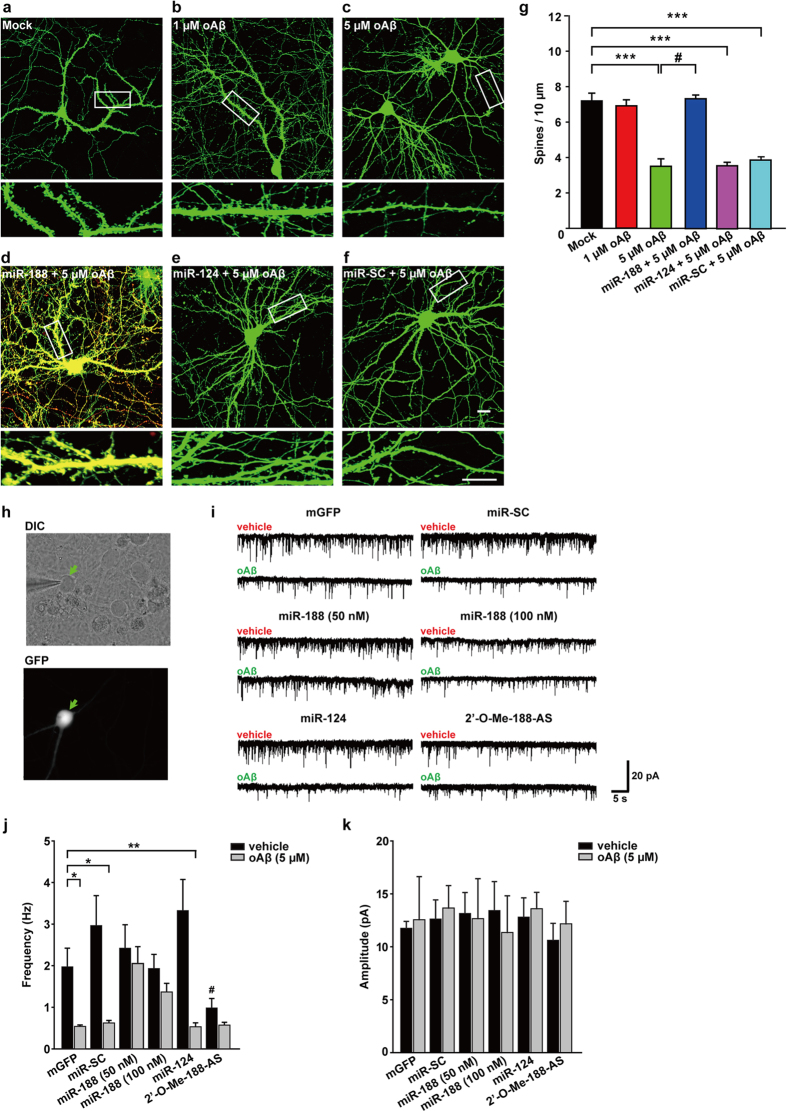
miR-188-5p restored the reduction in dendritic spine density and basal synaptic transmission induced by oligomeric Aβ_1-42_. (**a–f**) Representative confocal images of dendritic spines in rat primary hippocampal neurons at DIV 18–20 after treating with oligomeric Aβ_1-42_ (oAβ) for 24 h either alone or plus transfection with miR-188-5p (IRES-DsRed2), miR-124 or miR-SC oligonucleotides and the IRES-mGFP vector at DIV 10–12. The dendritic segment, outlined with a white box (upper), is magnified to delineate spine morphology (bottom). The scale bars indicate 20 μm (low-scaled panel) and 5 μm (magnified panel). (**g**) Treatment with oAβ (5 μM) for 24 h at DIV 17 induced a significant reduction in dendritic spine density (n = 9 neurons, vs. vehicle-treated group, n = 10 neurons, one-way ANOVA). However, transfection with the miR-188-5p oligonucleotide rescued the reduction in dendritic spine density induced by treatment with oAβ (n = 9 neurons, one-way ANOVA). Data are represented as the mean ± SEM. ***p < 0.001 compared to Mock; ^#^*p* < 0.01 compared to 5 μM oAb. (**h**) Representative images of the single whole-cell recording model used to measure transfected neurons (green arrow) at DIV 18–20. (**i**) Sample traces of mEPSCs recorded in rat primary hippocampal neurons treated with vehicle or 5 μM oAβ either alone or plus transfection with miR-SC, miR-124, miR-188-5p or 2′-O-Me-188-5p-AS oligonucleotides and the IRES-mGFP vector at DIV 10–12. Five minutes of representative mEPSC recordings were used to generate the cumulative distribution plot. (**j**) Bar graphs show the mean values of mEPSC frequencies of vehicle (black bar) and 5 μM oAβ-treated (gray bar) rat primary hippocampal neurons. Co-transfection of miR-188-5p (50 or 100 nM) with mGFP completely reversed the reduction of mEPSC frequency induced by 5 μM oAβ (n = 7 vs. vehicle, n = 8, *p* > 0.05). *p < 0.05, **p < 0.01 compared to mGFP-transfected control. (**k**) The mEPSC amplitudes of rat primary hippocampal neurons treated with each vehicle (black bar) were not altered compared to the 5 μM oAβ-treated neurons (gray bar). The statistical comparisons of synaptic currents were made using the Kolmogorov-Smirnov test. Statistical analysis was performed by an independent T test or nonparametric Mann-Whitney test; data represents the mean ± SEM. oAβ = oligomeric Aβ_1-42_.

We confirmed that the treatment with 5 μM oligomeric Aβ_1-42_ for 24 h induced a significant reduction in dendritic spine density at DIV 17 in rat primary hippocampal neuron cultures. However, transfection with the miR-188-5p restored the Aβ_1-42_-mediated reduction in dendritic spine density to the similar level of vehicle-treated group. The transfection of miR-scrambled (miR-SC) or miR-124, which is enriched in the brain^33^, but does not target Nrp-2, did not affect Aβ_1-42_-induced reduction in dendritic spine density (Fig. 3a–g). 9–10 neurons were analyzed for each group. The numbers of dendrites analyzed per neuron is 4.90 ± 0.64 (mock), 3.13 ± 0.30 (1 μM oligomeric Aβ_1-42_), 4.43 ± 0.37 (5 μM oligomeric Aβ_1-42_), 3.67 ± 0.41 (miR-188 + 5 μM oligomeric Aβ_1-42_), 4.67 ± 0.67 (miR-124 + 5 μM oligomeric Aβ_1-42_), 6.25 ± 0.84 (miR-SC + 5 μM oligomeric Aβ_1-42_). These results suggest that the decrease in miR-188-5p expression causes down-regulation of dendritic spine density.

Next, we recorded mEPSCs and analyzed the frequency and amplitude to measure basal synaptic transmission. A single whole-cell recording method was employed to record vehicle-treated or 5 μM Aβ_1-42_-treated rat primary hippocampal neurons transfected with IRES-mGFP plus microRNA oligonucleotides (Fig. 3h–k). mEPSC frequency in Aβ_1-42_-treated rat primary hippocampal neurons was significantly decreased compared to vehicle-treated neurons. However, in neurons treated with Aβ_1-42_ plus 50 nM or 100 nM miR-188-5p oligonucleotides, the reduction in mEPSC frequency was almost completely reversed back to control levels. Again, this was a miR-188-5p-specific effect as neurons treated with Aβ_1-42_ after co-transfection of miR-SC or miR-124 showed no effect on the attenuation of the Aβ_1-42_-mediated reduction in mEPSC frequency. Here, we also examined the effects of 2′-O-methyl (2′-O-Me) oligonucleotide for miR-188-5p (2′-O-Me-188-5p-AS), which serves as a miR-188-5p specific inhibitor on mEPSCs. First, the effects of 2′-O-Me-188-5p-AS on the level of miR-188-5p and miR-188-3p was examined, respectively. It was found that the level of miR-188-5p was significantly decreased by the treatment with 2′-O-Me-miR-188-5p AS for 6 days, while that of miR-188-3p did not show a significant change, indicating that 2′-O-Me-188-5p-AS serves as miR-188-5p specific inhibitor (Supplementary Fig. S2). Transfection of rat primary hippocampal neurons with 2′-O-Me-188-5p-AS reduced mEPSC frequency and showed no significant difference when compared to treatment of 2′-O-Me-188-5p-AS treated neurons with Aβ_1-42_ (Fig. 3j). The mEPSC amplitude was similar among any of the groups (Fig. 3k). These results demonstrate that miR-188-5p rescues the reduction in basal synaptic transmission induced by oligomeric Aβ_1-42_.

### miR-188-5p restored the synaptic dysfunction in 5XFAD mice

**Figure 4:**
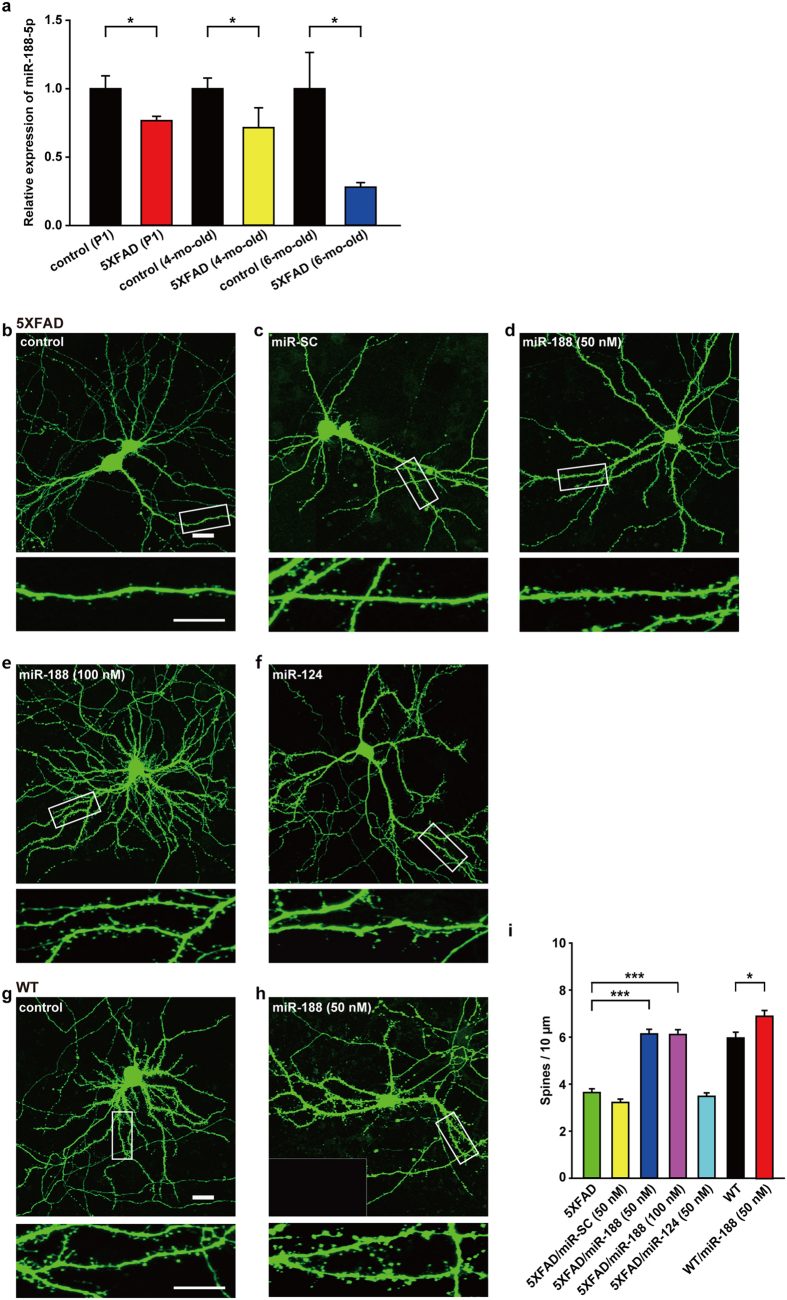
miR-188-5p rescued the reduction in dendritic spine density in primary hippocampal neurons cultured from P1 5XFAD mice. (**a**) miR-188-5p expression in the hippocampus was evaluated with RT-qPCR from P1, 4-month-old and 6-month-old 5XFAD mice. miR-188-5p was significantly down-regulated in the hippocampi of 5XFAD mice (n = 11 vs. age-matched controls, n = 7; at 4 months of age, n = 4 vs. age-matched controls, n = 5; at 6 months of age, n = 3, vs. age-matched controls, n = 4, Student t-test). U6 snoRNA was used as a reference control. The data represent the means a SEM. *p < 0.05 compared to age-matched wild-type mice. P1 = post-natal day 1. (**b–h**) Representative images of dendritic spines in primary hippocampal neurons of P1 wild-type and 5XFAD mice at DIV 18–20. The dendritic segment outlined with a white box (upper) is magnified to delineate the spine morphology (bottom) with a 4X optic zoom. The scale bars indicate 20- and 10-μm in the low- and high-magnification images, respectively. (**i**) A quantification of the spine densities (secondary dendritic spines 50–100 μm from the soma) at DIV 18–20 after transfection into primary hippocampal neurons at DIV 10–12. The dendritic spine densities of neurons from 5XFAD mice at DIV 18–20 were significantly reduced (n = 21 neurons, one-way ANOVA) compared to neurons from wild-type mice (n = 13 neurons). The addition of miR-188-5p to primary hippocampal neurons from 5XFAD mice significantly rescued the reduction in dendritic spine density in 5XFAD mice (n = 22 neurons, one-way ANOVA) compared to the neurons of untreated 5XFAD mice. Data are represented as the mean ± SEM. *p < 0.05, ***p < 0.001 compared to mGFP-transfected wild-type mice primary hippocampal neurons; ^#^*p* < 0.001 compared to mGFP-transfected 5XFAD mice primary hippocampal neurons.

It has been reported that adult 5XFAD mice show synaptic dysfunction in various brain regions^34^. We observed whether the expression of miR-188-5p is altered in 5XFAD mice compared with age-matched wild-type mice. RT-qPCR analysis showed that miR-188-5p was significantly down-regulated in the hippocampi of the 5XFAD mice at post-natal day 1 (P1), 4 months of age, and at 6 months of age (at P1, 0.77 ± 0.03, *p* = 0.027; at 4 months of age, 0.72 ± 0.15, *p* = 0.038; at 6 months of age, 0.28 ± 0.03, *p* = 0.014; Fig. 4a).

The dendritic spine densities of the primary hippocampal neurons from 5XFAD mice at DIV 18–20 were significantly decreased by 59.74% compared to the neurons from wild-type mice (*p* < 0.001; Fig. 4b,g,i). Conversely, the transfection with miR-188-5p oligonucleotide into the neurons significantly rescued the reduction in dendritic spine density in 5XFAD mice compared to only mGFP-transfected neurons from 5XFAD mice (*p* < 0.001; Fig. 4d,e,i). 9–10 neurons were analyzed for each group. The numbers of dendrites analyzed per neuron is 4.52 ± 0.31 (5XFAD), 4.41 ± 0.35 (5XFAD/miR-SC), 4.24 ± 0.34 (5XFAD/miR-188 50 nM), 3.75 ± 0.27 (5XFAD/miR-188 100 nM), 4.00 ± 0.36 (5XFAD/miR-124), 5.46 ± 0.62 (wild-type) or 3.29 ± 0.29 (wild-type/miR-188 50 nM).

Here, we showed that primary hippocampal neurons from 5XFAD mice exhibited a reduction in dendritic spine density compared to neurons from wild-type mice. A previous report demonstrated that neurons prepared from Tg2576 mice exhibited abnormal morphologies and lower spine density compared to neurons from wild-type control animals^35^. This result is because the higher level of Aβ is formed in neuron culture as shown in the previous paper^36^. Moreover, we confirmed that this was a miR-188-5p-specific effect as no significant rescue was observed after transfection with either miR-SC or miR-124 (Fig. 4b,c,f,i).

### miR-188-5p rescued the memory deficits in 5XFAD mice

**Figure 5:**
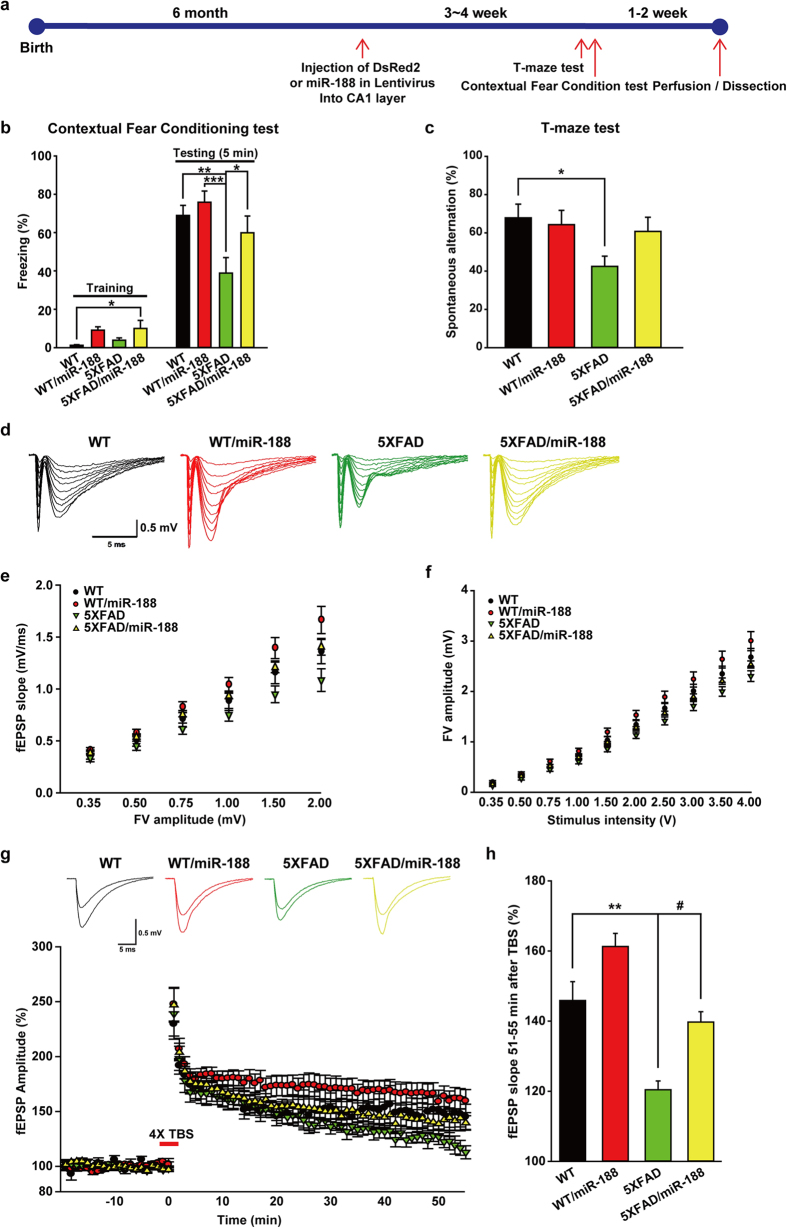
miR-188-5p rescued the memory deficits by restoring synaptic dysfunction in 5XFAD mice. (**a**) Experimental schedule for miR-188-5p overexpression in wild-type and 5XFAD mice. Mice were trained with 3 foot shocks (0.7 mA, 2 sec) for contextual fear conditioning. (**b**) 5XFAD mice showed significantly lower levels of contextual freezing than wild-type mice when tested 1 day after training. 5XFAD/miR-188-5p mice showed complete rescue of freezing, similar to the wild-type mice (n = 8–10 mice per group, one-way ANOVA). (**c**) 5XFAD mice showed significantly reduced levels of spontaneous alternation performance in the T-maze (n = 14, one-way ANOVA), compared to wild-type mice (n = 10). Viral-mediated expression of miR-188-5p restored the reduction in spontaneous alternation performance shown in 7-month-old 5XFAD mice (n = 11, one-way ANOVA). *p < 0.05, **p < 0.01 and ***p < 0.001 compared to control virus injected 5XFAD mice; ^*#*^*p* < 0.05 compared to control virus injected wild-type mice. (**d–f**) Restoration of basal synaptic transmission in 5XFAD mice by miR-188-5p expression. (**d**) Sample traces of synaptic responses at the SC-CA1 synapse with various stimulation intensities in each group. (**e**) The relationship between fEPSP slope and FV amplitude in each group (n = 1 ~ 6 slices from 4 mice for each group). (**f**) The FV amplitudes were plotted against stimulation intensities. (**g**,**h**) Impaired LTP in 5XFAD mice was recovered by miR-188-5p expression. (**g**) Representative traces of fEPSP responses during baseline and 51–55 min after 4X TBS in each group (up). LTP induced by 4X TBS at SC-SC1 synapses (bottom). Each point represents mean fEPSP slope normalized to the average baseline response before TBS. (**h**) Summary of the magnitude of mean LTP during 51–55 min after TBS relative to baseline in each group (wild-type, n = 1–6 slices from 4 mice for each group ; wild-type/188-5p, n = 12; 5XFAD, n = 8; 5XFAD/188-5p, n = 10). All data represent the mean ± SEM of mice pooled from 4 mice for each group. **p < 0.01 compared to control virus injected wild-type mice, ^*#*^*p* < 0.0001 compared to control virus injected 5XFAD mice by non-parametric Mann-Whitney test. TBS = theta-burst stimulation, FV = fiber volley.

To further explore whether the reduction in miR-188-5p expression observed in the brains from AD patients and 5XFAD mice regulates cognitive function due to changes in synaptic structure and basal synaptic function, we expressed miR-188-5p subcloned in a lentiviral vector in the CA1 region (Fig. 5a) and we confirmed miR-188-5p expression in the hippocampus of naive mice 3 weeks after stereotaxic injection (Supplementary Fig. S3). A schematic diagram of the experimental procedure is shown in Fig. 5a. To examine if miR-188-5p expression ameliorates the deficits in hippocampus-dependent learning and memory observed in 7-month-old 5XFAD mice, we first performed a contextual fear conditioning test, in which mice learn to associate a distinct context with aversive footshocks^37^. Wild-type mice exhibited a robust conditional fear response, which was assessed by freezing when returned to the conditioning chamber after training. 5XFAD mice (38.48 ± 8.04%, *p* = 0.004) exhibited a strongly lower percentage of freezing compared with wild-type controls (68.27 ± 5.23%). However, viral-mediated expression of miR-188-5p in 5XFAD mice significantly rescued freezing behavior. These mice showed higher levels of freezing (59.33 ± 8.67%, *p* = 0.047), similar to wild-type controls (Fig. 5b).

Next we measured spatial working memory in 5XFAD mice using T-maze. These mice showed significantly reduced levels of spontaneous alternation performance (37.50 ± 5.10, *p* = 0.002), compared to wild-type mice (67.50 ± 5.34; Fig. 5c). Expression of miR-188-5p reversed the reduction in spontaneous alternation performance (56.82 ± 7.61, *p* = 0.037).

To investigate the synaptic mechanisms underlying the enhancement of learning and memory by miR-188-5p, we first examined basal synaptic transmission at the Schaffer collateral (SC)-CA1 synapses through fEPSP recording. Consistent with previous observations^38^, 5XFAD mice showed clear synaptic deficits (Fig. 5d). The relationship between the fEPSP slope and the fiber volley (FV) amplitude was significantly reduced in 5XFAD mice compared with wild-type control mice (Fig. 5e). However, FV amplitude stimulation intensity ratios were not different in all experimental groups (Fig. 5f). These results imply that reduced synaptic transmission in 5XFAD mice might stem from deleterious effects of Aβ on postsynaptic compartments rather than a reduced number of active presynaptic fibers (Figs 3 and 4). Unexpectedly, viral-mediated expression of miR-188-5p in the 5XFAD CA1 neurons significantly increased synaptic strength. The fEPSP slope to FV amplitude ratios of 5XFAD/miR-188-5p mice were almost indistinguishable from those of control mice.

We next examined the effect of miR-188-5p expression on synaptic LTP. Although repeated trains of theta-burst stimulation (4XTBS) induced synaptic potentiation at SC-CA1 synapses in all experimental groups (Fig. 5g), we could observe genotype-specific differences in magnitude and duration of potentiation. This observation is consistent with several previous studies^38^,^39^. While control slices exhibited stable enhancement of synaptic transmission (147.02 ± 5.48% at 50 min after 4 X TBS), LTP in 5XFAD slices gradually decreased in magnitude toward baseline during the recording (121.5895 ± 2.4389% at 50 min after 4 X TBS, *p* < 0.01). Notably however, impaired LTP in 5XFAD mice almost completely recovered to a normal magnitude with miR-188-5p replenishment in CA1 neurons and no significant difference in LTP was detected between 5XFAD/miR-188-5p and wild-type mice (141.50 ± 2.93% at 50 min after 4X TBS; Fig. 5g,h). These results suggest that miR-188-5p replenishment rescues synaptic dysfunction in 5XFAD mice, and that dysregulation of an activity-regulated miR-188-5p might be associated with memory deficits in 5XFAD mice.

### CREB regulates miR-188 expression

**Figure 6:**
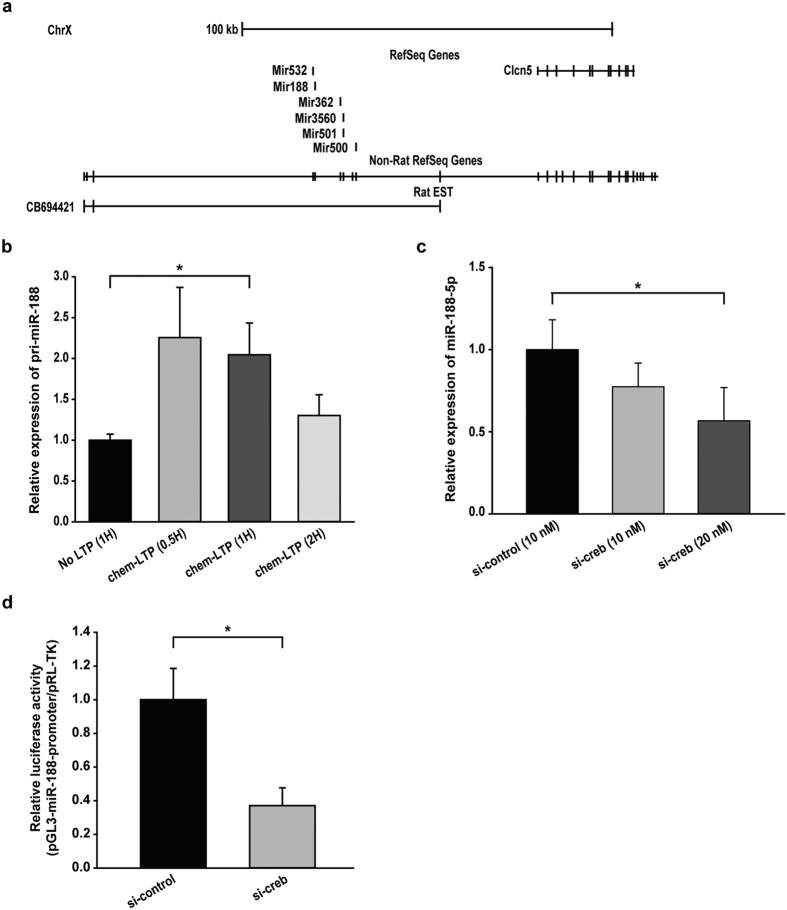
CREB regulates miR-188 expression. (**a**) The location of miR-188, CB694421, and Clcn5 is shown using the USCS genome browser. (**b**) RT-qPCR was performed to investigate the changes in pri-miR-188 by chemical LTP induction. Pri-miR-188 level was significantly increased in rat hippocampal slices by chemical LTP induction (No; n = 3, 0.5 h; n = 3, 1 h; n = 3, 2 h; n = 3, one-way ANOVA). *p < 0.05 compared to No LTP. (**c**) RT-qPCR was performed to investigate the changes in miR-188-5p followed by *Creb* knockdown. Knockdown of *Creb* using Creb siRNAs resulted in significant downregulation of mature miR-188-5p levels in rat primary hippocampal neurons (si-creb 10 nM; n = 4, si-creb 20 nM; n = 4, compared to control; n = 3, one-way ANOVA). *p < 0.05 compared to control siRNAs. (**d**) miR-188 promoter activity was significantly reduced by *Creb* knockdown. (n = 3, compared to control siRNAs, Student’s t-test) in rat primary hippocampal neuron cultures. *p < 0.05 compared to control siRNAs.

Here we set out to uncover the regulatory mechanism for miR-188 expression. We first tested whether LTP induction increases transcription levels of miR-188. We found that levels of miR-188 primary transcript (pri-miR-188) were significantly increased in rat hippocampal slices by chemical LTP induction (Fig. 6b), suggesting that miR-188 levels are likely up-regulated at the transcriptional level.

We then tried to identify transcription factors critical for LTP-mediated upregulation of miR-188-5p. To this end, we determined the genomic locations of *MIR188* gene in the rat genome via the UCSC genome browser, and found that *MIR188* gene is located at approximately 50 kb upstream from the transcriptional start site of the *Clcn5* gene on the X-chromosome (Fig. 6a). Interestingly, we also found that there was an expressed sequence tag (EST) containing *MIR188* gene (CB694421), suggesting that CB694421 might serve as pri-miR-188. Given this finding, we performed analysis on transcription factor binding sites in the promoter region of CB694421 using TRANSFAC. We found one CREB binding site within 2 kb upstream from the 5′ end of CB694421. Because the role of CREB in synaptic plasticity has been well established, we tested whether CREB is involved in the regulation of miR-188 expression. Knockdown of *Creb* using small interfering CREB RNA (si-CREB) indeed resulted in significant down-regulation of mature miR-188-5p levels in rat primary hippocampal neurons (56.63 ± 20.22%, *p* = 0.017; Fig. 6c), suggesting that CREB can regulate miR-188-5p expression. We also performed miR-188 promoter luciferase activity assay using rat primary hippocampal neuron cultures. The miR-188 promoter activity was suppressed by CREB knockdown (37.03 ± 10.66%, *p* = 0.015; Fig. 6d) compared to control siRNAs. Taken together, these data suggest that LTP induction increases miR-188 levels potentially through CREB activation.

## Discussion

In the present study, we aimed to investigate the contribution of miR-188-5p to AD, which is reduced in the disease. Aβ diminished the expression of miR-188-5p in rat primary hippocampal neurons. The addition of miR-188-5p rescued the oligomeric Aβ_1-42_-mediated reduction in dendritic spine density in primary hippocampal neurons and neurons from 5XFAD mice. In addition, the decrease in the frequency of mEPSCs induced by oligomeric Aβ_1-42_ was restored by the addition of miR-188-5p in primary hippocampal neurons. Furthermore, we have shown that the impairments in cognition and fEPSPs observed in 7-month-old 5XFAD mice were ameliorated via the viral-mediated expression of miR-188-5p in the hippocampus.

LTP is believed to be a synaptic mechanism underlying the storage of long-term memories in the brain^15^,^40^. Therefore, enhancement of LTP by miR-188-5p expression explains the improved behavioral outcomes in 5XFAD mice. In line with this idea, decrease of miR-188-5p might be one of the possible mechanisms for the cognitive deficits in AD patients and 5XFAD mice. In addition, exogenous expression of miR-188-5p increased synaptic strength in 5XFAD mice. Considering the role of miR-188-5p in the regulation of dendritic spine density, this effect might have originated from restored spine density in 5XFAD neurons by miR-188-5p overexpression. Although molecular mechanisms underlying spine formation and synaptogenesis are relatively uncharacterized^41^, our results suggest that miR-188-5p increases dendritic spine density through the downregulation of Nrp-2^14^.

Recently it has been reported that miR-188-3p targets β site cleavage enzyme (BACE1) and miR-188-3p expression was significantly down-regulated both in the brains of AD humans and 5XFAD mice and that miR-188 expression is regulated by 2-arachidonoyl glycerol or peroxisome proliferator-activated receptor-γ (PPARγ) agonists^42^. In the present study, we investigated the pathophysiological significance of miR-188-5p in AD by adding miR-188-5p oligonucleotides or lenti-viral vector expressing miR-188-5p specifically.

The roles for miR-188 were also reported in cancer cells. Overexpression of miR-188 inhibits cell proliferation, tumor colony formation and G1/S cell cycle transition in human nasopharyngeal carcinoma CNE cells by inhibiting CCND1, CCND3, CCNE1, CCNA2, CDK4 and CDK2^43^. Furthermore, miR-188 regulates the age-related switch between switch osteogenesis and adipogenesis of bone marrow mesenchymal stem cells by targeting histone deacetylase 9 (HDAC9) and RPTOR-independent companion of MTOR complex 2 (RICTOR)^44^.

In this study, we showed that CREB can regulate the expression of miR-188 in rat primary hippocampal neurons. Based on the fact that miR-188 sequences are embedded in the CB694421 EST, we hypothesized that the CB694421 EST might serve as pri-miR-188, and found one CREB binding site within 2 kb upstream of the 5′ end of CB694421. Chemical induction of LTP increased levels of pri-miR-188 in rat hippocampal slices (204.59 ± 38.87%, *p* = 0.034; Fig. 6b).

A given microRNA may have multiple (up to several hundred) predicted gene targets, and ~60% of mRNAs have predicted binding sites for several microRNAs in their 3′UTRs. Although the mechanisms that regulate the expression of microRNA genes subtly remain veiled, it seems clear that microRNAs exert a profound impact on gene regulatory networks and regulate development, homeostasis, and diseases such as neurodegenerative diseases^45^.

In AD, the dysfunction or dysregulation of microRNAs is reported to be related to the pathogenesis of AD. The miR-20a family and miR-101, which target APP, are down-regulated in AD patients^46^,^47^. Other microRNAs, including miR-9, regulate one of the secretases that produces Aβ, BACE1, and their expression was also reduced in AD patients^48^,^49^. Recently, miR-125b, which is elevated in AD, is reported to induce tau hyperphosphorylation and cognitive deficits in AD by targeting Bcl-W, DUPS6 and PPP1CA^50^. As mentioned above, the dysregulation of microRNA expression is related to the pathogenesis of AD and other neurological diseases including Parkinson’s disease^46^,^51^,^52^,^53^.

In conclusion, our data suggest that reduction of miR-188-5p is a key regulator of aberrant synapse elimination in AD. This may lead to cognitive deficits, accompanied by Aβ-mediated synapse death signal pathways and synaptic dysfunction in the hippocampus. A schematic diagram showing a possible relationship among miR-188-5p, Nrp-2, BDNF and CREB in normal condition and AD context was shown in Supplementary Fig. S4. Taken together with the previous study^42^, dysregulation of miR-188-3p and -5p expressions contribute to the pathogenesis of AD by distinct mechanisms.

## Methods

### Animals

All animal experimental procedures were approved by the Animal Care Committee of Seoul National University (Approval number: SNUIBC-080919-1). Transgenic mice with 5XFAD mutations were purchased from Jackson Laboratories (strain: B6SJL-Tg [APPSwFlLon, PS1*M146L*L286V] 6799Vas/J) and maintained by crossing hemizygous transgenic mice with B6SJL F1 mice. And wildtype male C57BL/6 mice (25–30 g) were supplied by Koatech (Pyeongtaek, Korea). Animal treatment and maintenance were performed in accordance with the Animal Care and Use Guidelines of Seoul National University, Seoul, Korea.

### Monomeric and oligomeric Aβ_1-42_ preparation and BDNF preparation

Monomeric and oligomeric Aβ were prepared as previously reported^54^,^55^. Synthetic Aβ_1-42_ peptide (American Peptide, Sunnyvale, CA, USA) was dissolved to 1 mM in 100% hexafluoroisopropanol (HFIP, Sigma Chemical Company, MO, USA). The solution was allowed to evaporate for 2 h in a Speed Vac (SPD2010, Thermo Savant, NY, USA). The resulting peptide film was stored at −20 °C or immediately resuspended in dimethyl sulfoxide (DMSO, Sigma Chemical Company) to produce a 1 mM solution. We used this for mAβ_1-42_ without any pre-cooling or freezing step. Then, to prepare oligomeric Aβ_1-42_, this solution was diluted to 100 μM in phenol red-free Ham’s F-12 medium (Life Technology, NY, USA) and incubated for 12 h at 4 °C. Human recombinant BDNF was purchased from ProSpec-Tany TechnoGene (#CYT-207, Rehovot, Israel). Lyophilized BDNF was reconstituted using sterile water.

### Primary hippocampal neuron culture

Primary hippocampal neuron cultures were prepared from E18-19 pregnant Sprague-Dawley (SD) rats or from P1 5XFAD mice by dissociating with 0.25% trypsin and plated onto coverslips coated with poly-L-lysine. Neurons were grown in Neurobasal medium (Gibco, CA, USA) supplemented with B27 (Gibco, CA, USA), 2 mM GlutaMAX-I supplement (Gibco, CA, USA) and 100 μg/ml penicillin/streptomycin (Gibco, CA, USA) at 37 °C in a humidified environment of 95% O_2_/5% CO_2_.

### DNA constructs and oligonucleotides

IRES-mGFP vector was a generous gift from Dr. A. L. Kolodkin, The Johns Hopkins University School of Medicine, Baltimore, MD. Expression vectors for miR-188-5p (miRBase Accession No. MIMAT0005301) were prepared by introducing synthesized oligonucleotides corresponding to the miR-188-5p sequences and complementary sequences into pLL3.7-DsRed2 vector. All constructs were sequenced using an ABI310 Sequencer. Oligonucleotides used are detailed in Supplementary Experimental Procedures.

### Human AD brains

Paraffin-embedded brain stocks and the frozen tissues from 69 to 98 years old-AD and age-matched control subjects were obtained from the Netherlands Brain Bank (http://www.brainbank.nl/about-us/the-nbb/). Tissues from AD patients were diagnosed by neuropathological evidence using the criteria for Braak & Braak stage V or VI. The neuropathological diagnosis for non-demented controls consisted of the neuropathological criteria for classification as Braak & Braak stage 0 or I. Coronal sections (4 μm) were cut through the hippocampus and processed for immunohistochemistry. For western blot analysis, frozen brain tissues were used. All experimental procedures were performed in accordance with ‘the Guidelines of the Ethics Committee at Seoul National University’.

### RT-qPCR

Total RNA or specifically the small RNA fraction was extracted by miRNeasy Mini kit (cat no. 217004, Qiagen, CA, USA) or NucleoSpin microRNA kit (cat no. 740971, Macherey-Nagel, Duren, Germany), and 0.5-1.0 μg RNA was processed for cDNA synthesis using miScript PCR Starter Kit (cat no. 218193, Qiagen, CA, USA) according to the manufacturer’s instructions. Primers used are described in Supplementary Experimental Procedures. To quantify the microRNA expression levels, SYBR Green microRNA assay-based RT-qPCR (using miScript PCR Starter Kit) was performed on a 7500 Fast Real-Time PCR systems (Applied Biosystems, CA, USA), using the *ΔΔ*Ct method. ROX was utilized as an endogenous reference to standardize the microRNA expression levels. All of the data were normalized by the snRNA RNU6B or 5S rRNA. The primers used are detailed in Supplementary Experimental Procedures.

### Immunohistochemistry

Human AD or age-matched control brains were incubated in 10% neutral buffered formalin for 48 h and then dehydrated and embedded in paraffin. Prior to immunostaining, slides were deparaffinized by oven heating and immersion in xylene. After dehydration through graded alcohols and water, tissue slices were immunostained overnight with a primary antibody against Nrp-2 (Cell Signaling Technology, MA, USA) at 1:50, followed by Alexa Fluor 488-conjugated secondary antibodies (Molecular Probes, CA, USA) at 1:100 After three washes in permeabilization buffer and a wash in PBS, cells were mounted on microscope slides in mounting medium (DAKO, CA, USA). Confocal microscopy was performed using an LSM 510 (Carl Zeiss, Jena, Germany).

### Western blot

Whole cell lysates or hippocampi extract samples were electrophoresed on a denaturing 10–15% SDS-PAGE gels and transferred to PVDF membranes (Millipore, MA, USA). Each membrane was probed with primary antibodies; Nrp-2 (Cell Signaling Technology, MA, USA) at 1:2,000, GAPDH (Santa Cruz Biotechnology, TX, USA) at 1:5,000. After washing, the membrane was incubated for 1 h at room temperature with Goat anti-Rabbit IgG (H + L), HRP (Molecular Probes, NY, USA). The HRP signals were visualized using an enhanced chemiluminescent (ECL) substrate (Thermo Fisher Scientific, IL, USA).

### Dendritic spine density analysis

Primary hippocampal neuron cultures from SD rat (E18–19) were transfected with 3 μg -IRES-mGFP, and with or without pLL3.7-miR-188-IRES-DsRed plasmid in 18 mm Φ in 12-well plates. The number of dendritic spines was evaluated at DIV 18. Fluorescent images were acquired by confocal microscopy (LSM 510, Carl Zeiss, Jena, Germany) using identical settings for all samples. Spines were counted on 20–40 μm segments of secondary dendrites extending at least 40–80 μm beyond the cell body (soma). 3–4 segments from each neuron were quantified. Primary hippocampal neuron cultures (DIV 10–12) from wild-type and 5XFAD P1 mice were transfected with one of the following combinations: 1) IRES-mGFP control vector alone; or 2) IRES-mGFP control vector plus the miRNA mimic oligonucleotides. The number of dendritic spines was evaluated at DIV 18–20.

### Whole-cell patch clamp studies

Whole cell voltage-clamp was performed with a MultiClamp 700B amplifier (Molecular Devices, CA, USA). The series resistance (10–30 MΩ) was monitored in all experiments. The membrane potential was held at −70 mV during the recording. The frequency and amplitude of the mEPSCs were analyzed with the Mini Analysis program (Synaptosoft, NJ, USA). The noise level was below 5 pA, and 7 pA and was typically used as the threshold for mEPSC events. Five minutes of representative mEPSC recordings were used to generate the cumulative distribution plot. Recordings are detailed in Supplementary Experimental Procedures.

### Hippocampal slice preparation and chemical LTP induction

Acute hippocampal slices were prepared from 4- to 5-week-old (90~110 g) male SD rat brains. Briefly, brains were rapidly removed and coronal brain slices (400 μm) containing the hippocampus, were cut on a Vibratome (Leica, Germany) in ice-cold aCSF [119 mM NaCl, 2.5 mM KCl, 1 mM MgSO_4_, 2.5 mM CaCl_2_, 1.25 mM NaH_2_PO_4_, 26 mM NaHCO_3_ and 10 mM glucose] that was bubbled with 95% O_2_/5% CO_2_ and adjust to pH 7.4. After a 1.5 h recovery at 27 °C, an individual slice was transferred to a submerged recording chamber and continuously superfused with oxygenated aCSF at a rate of 2.5–3 ml/min at 33 ± 1 °C.

LTP was introduced as previously described^56^, and was then recorded in basal bath solution for 2 h. Electrical stimulation intensity was normalized to the value of the basal fiber volley (FV) amplitude. Then, average responses (mean ± SEM) were expressed as the percentage of basal fEPSP amplitude. Recordings are detailed in Supplementary Experimental Procedures.

### Behavioral tests

Animals used were 7-month-old adult male mice. Before testing, mice were habituated to the testing room for 1 h. (see Supplementary Experimental Procedures).

### Luciferase activity assay

The 1992 bp rat miR-188 3′-UTR containing the putative cAMP responsive element was PCR-amplified from rat genomic DNA by using the forward 5′-tcttacgcgtgctagccctggcattttaatttagctc-3′ and reverse 5′-ccggaatgccaagctt gtttgcctttacctgtcac-3′ primers and the DNA fragment was cloned into the Nhe I and HindIII sites on the 5′ end of the luc^+^ gene on the pGL3-basic vector. Briefly, the primary hippocampal neurons were cotransfected with pGL3-miR-188-promoter vector or pGL3-basic vector, pRL-Tk Renilla luciferase reporter vector (Promega, WI, USA) and 20 nmol/L of small RNAs (Silencer Select pre-designed siRNA or Silencer Select Negative Control #1 siRNA, Ambion, Life Technologies, Carlsbad, CA, USA) using Lipofectamine 3000 (Life Technologies). The luciferase activity was determined 72 h post transfection and the reporter assay was performed according to the manufacturer’s protocol (Dual-Glo Luciferase Assay System, Promega). Firefly luciferase activity (mean ± SEM) was normalized to renilla luciferase and expressed as a percentage of the control.

### LDH assay

Primary hippocampal neuron cultures from SD rat (E18–19) were plated in 24-well plates and incubated at 37 °C. On DIV-17, the neurons were treated with vehicle or 5 μM oAβ. After the treatment for 24 h, the cell toxicity was assessed using the CytoTox 96 nonradioactive cytotoxicity assay kit (Promega, WI, USA) according to the manufacturer’s protocol. Briefly, for quantitative analysis, a 50 μl aliquot was transferred from each well to a 96 well plate. Then, 50 μl of the reagent was added to each well, and the reactions were incubated for 30 min at room temperature in the dark. After adding 50μl of stop solution to each well, the fluorescence intensity was measured at 492 nm. Absorbance was measured using a TECAN Infinite M200 plate reader (TECAN, Männedorf, Switzerland). The obtained values were normalized to those of the completely lysed control. All experiments were performed in biological triplicate.

### Statistical analysis

The data are represented as the means ± standard error of the mean (SEM) values. Student’s t-test, non-parametric Mann-Whitney U test and a one-way ANOVA using post-hoc comparisons (IBM SPSS Statistics 20, IL, USA) were used to determine statistical significance. The results were considered to be statistically significant if *p* < 0.05.

## Additional Information

**How to cite this article**: Lee, K. *et al*. Replenishment of microRNA-188-5p restores the synaptic and cognitive deficits in 5XFAD Mouse Model of Alzheimer’s Disease. *Sci. Rep.* **6**, 34433; doi: 10.1038/srep34433 (2016).

## Supplementary Material

Supplementary Information

## Figures and Tables

**Table 1:** Information on the control subjects and Alzheimer’s disease patients.

Name	NBB Number	Autopsy Number		Code	Sex	Age	Anatomical region
cerebral cortex-control #1	00–032	S00/059	100	K1	F	78	medial frontal gyrus
cerebral cortex-control #2	99–100	S99/214	100	K1	M	79	medial frontal gyrus
cerebral cortex-control #3	02–024	S02/055	300	GFM2	F	75	medial frontal gyrus
cerebral cortex-Alzheimer’s disease #1	98–015	S98/028	234	A2	F	87	medial frontal gyrus
cerebral cortex-Alzheimer’s disease #2	00–054	S00/115	300	GFM1	M	59	medial frontal gyrus
cerebral cortex-Alzheimer’s disease #3	03–017	S03/042	300	GFM2	M	67	medial frontal gyrus
cerebral cortex-Alzheimer’s disease #4	04–068	S04/232	300	GFM2	F	72	medial frontal gyrus
cerebral cortex-Alzheimer’s disease #5	00–091	S00/194	300	GFM2	F	76	medial frontal gyrus
hippocampus-control #1	00–032	S00/059	100	D3	F	78	hippocampus
hippocampus-control #2	99–100	S99/214	100	D3	M	79	hippocampus
hippocampus-control #3	02–024	S02/055	300	HIP2	F	75	hippocampus
hippocampus-Alzheimer’s disease #1	98–015	S98/028	234	B1	F	87	hippocampus
hippocampus-Alzheimer’s disease #2	00–054	S00/115	300	HIP1	M	59	hippocampus
hippocampus-Alzheimer’s disease #3	03–017	S03/042	300	HIP2	M	67	hippocampus
hippocampus-Alzheimer’s disease #4	04–068	S04/232	300	HIP3	F	72	hippocampus
hippocampus-Alzheimer’s disease #5	01–076	S01/173	300	HIP3	M	75	hippocampus

A human sample stock list from the cerebral cortices and hippocampi of control subjects and AD patients. NBB = Netherlands brain bank.
