# Supplementary material for: Replenishment of microRNA-188-5p restores the synaptic and cognitive deficits in 5XFAD Mouse Model of Alzheimer’s Disease
Source: Sci Rep. 2016 Oct 6;6:34433. doi: 10.1038/srep34433 (PMC5052619; doi:10.1038/srep34433)
Supplement: Supplementary Information [file srep34433-s1.doc]

**Replenishment of microRNA-188-5p restores the synaptic and cognitive deficits in 5XFAD Mouse Model of Alzheimer's Disease**

Kihwan Lee^1,2,*^, Hyunju Kim^1,*^, Kyongman An^3^, Oh-Bin Kwon^3^, Sungjun Park^3^, Jin Hee Cha^4^, Myoung-Hwan Kim^4^, Yoontae Lee^3^, Joung-Hun Kim^3^, Kwangwook Cho^5,6^, Hye-Sun Kim^1,7^

**
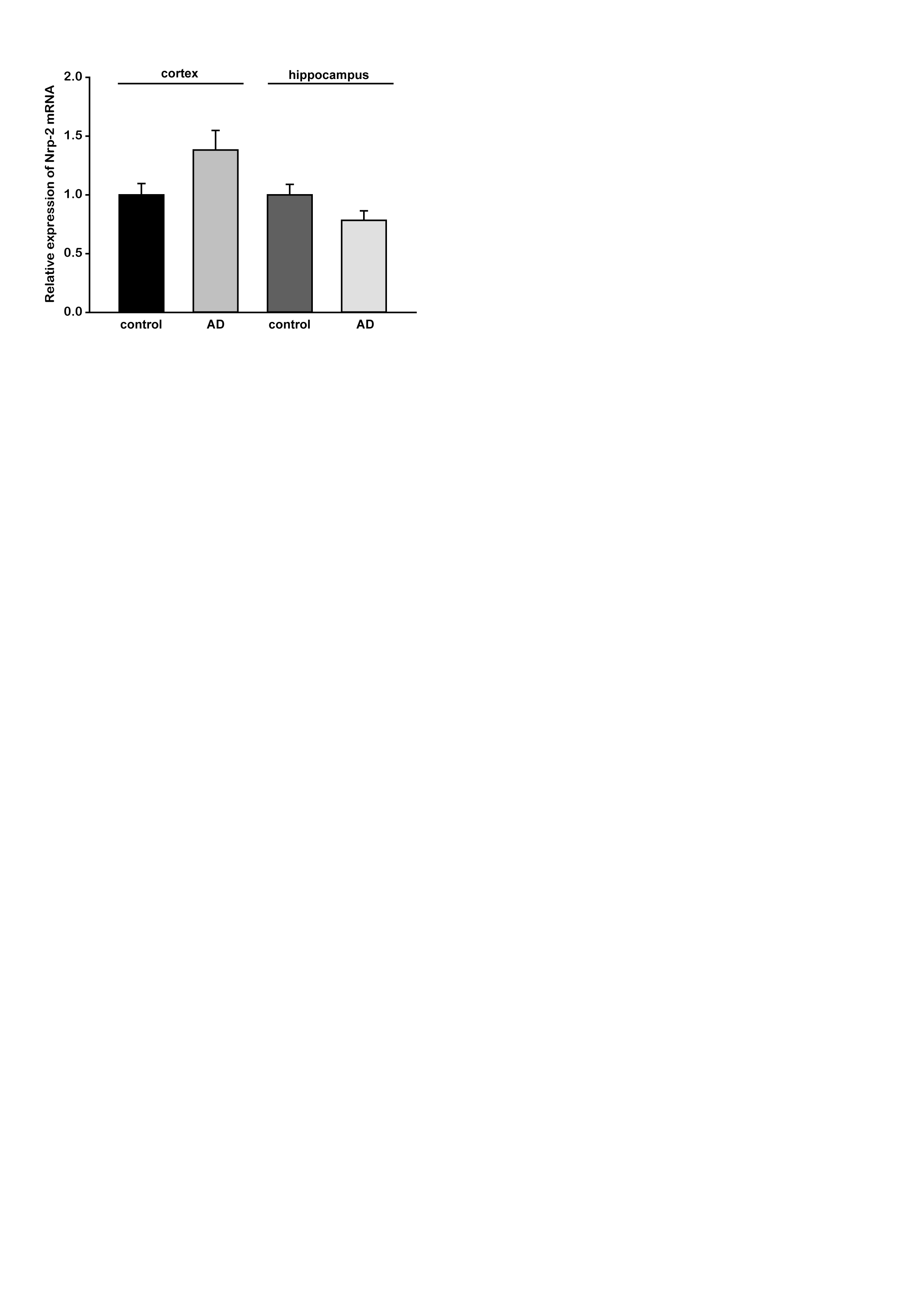
**

**Supplementary Figure 1. mRNA levels of Nrp-2 in AD patients were not different from age-matched control subjects.** The mRNA levels of Nrp-2 were evaluated with RT-qPCR. A significant difference was not observed between age-matched control subjects and AD patients. Data are represented as the mean ± SEM.


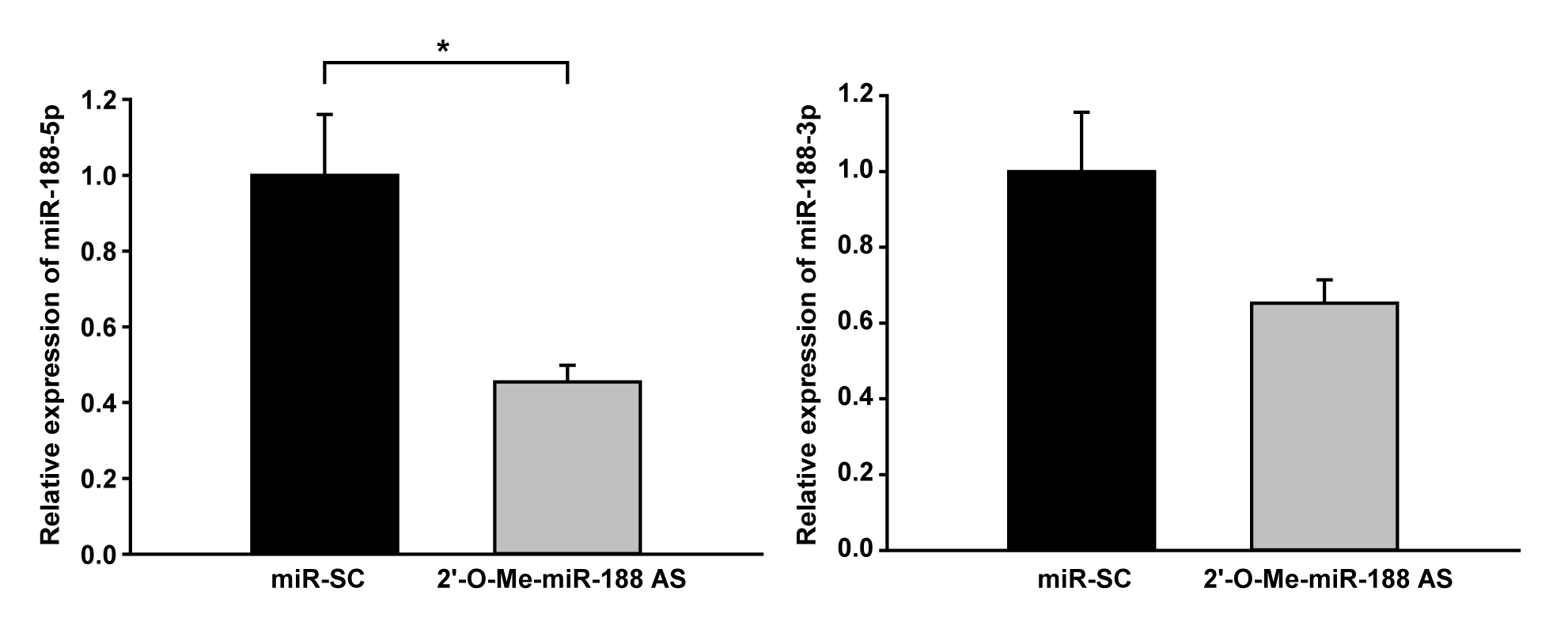


**Supplementary Figure 2. 2’-O-Me-miR-188-5p AS treatment specifically reduced miR-188-5p specifically in rat primary hippocampal neuron cultures.** The expression level of miR-188-5p and miR-188-3p was evaluated with RT-qPCR in rat primary hippocampal neuron cultures. The level of miR-188-5p was significantly decreased (0.45 ± 0.04, n = 5, 5, p = 0.025, Student’s t-test) by 2’-O-Me-miR-188-5p AS treatment while that of miR-188-3p did not show a significant change.

**
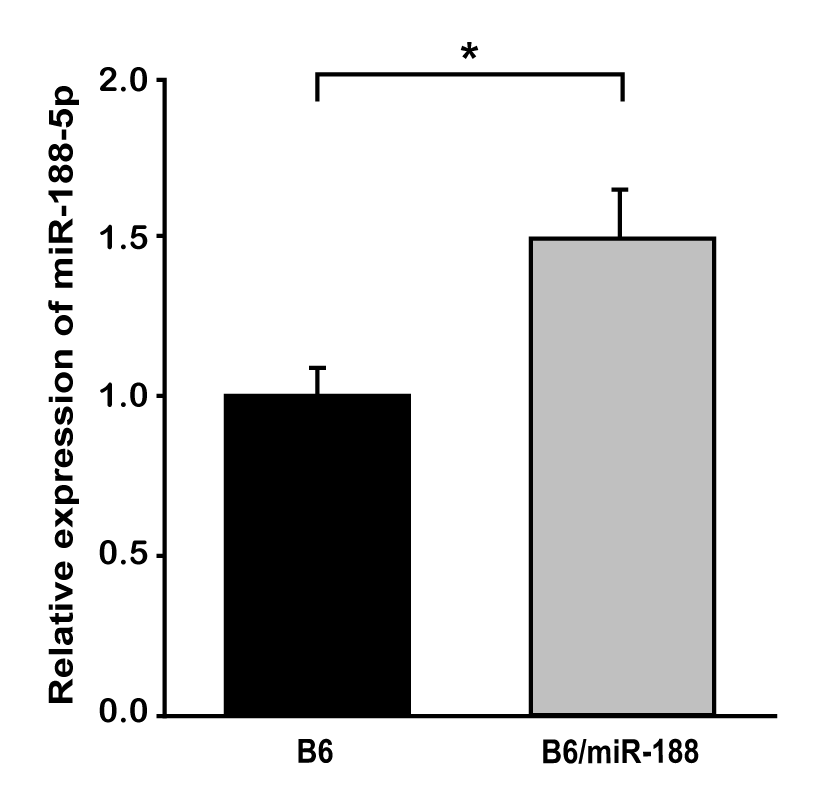
**

**Supplementary Figure 3. Viral-mediated expression of miR-188 in the hippocampus of C57BL/6 mice.** The levels of miR-188 were evaluated with RT-qPCR. miR-188 expression was significantly increased (1.49 ± 0.15, n = 4, 6, p = 0.033, Student’s t-test). Data are represented as the mean ± SEM.


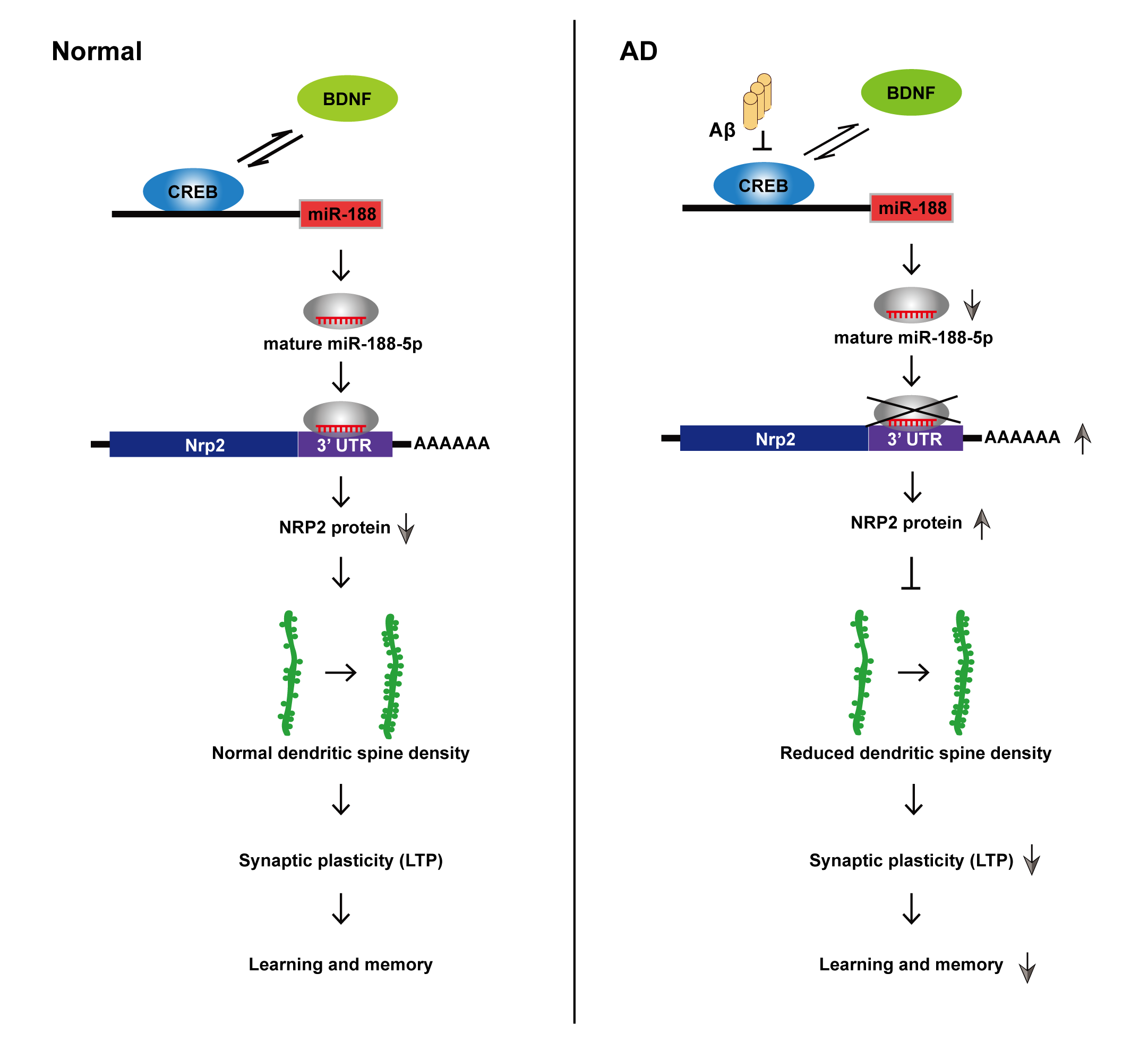


**Supplementary Figure 4. A schematic diagram for possible upstream and downstream signaling pathways of miR-188-5p.** This diagram shows a possible relationship among miR-188-5p, Nrp-2, BDNF and CREB in normal condition and AD context.

**Supplementary Experimental Procedures**

**RT-qPCR primers**

The primers for miR-188-5p (cat no. MS00001757, miScript Primer Assays), miR-188-3p (cat no. MS00011312, miScript Primer Assays), snRNA RNU6B (RNU6-2), which were provided in miScript PCR Starter Kit, were obtained from Qiagen (CA, USA). Primers for pri-miR-188 (5'-TGTGGCTATCTTGCTGCCC-3', 5'-GAGTCATTCTCCTTCCCACC-3'), and Nrp-2 (5'-AGAAGCCCGCTGAGATCT-3', 5'-CTCTCTGTCAAAAATGGATAT-3') were obtained from Bioneer (Daejeon, South Korea).

**Oligonucleotides**

The sequence of miR-188 mimic is 5’-CATCCCTTGCATGGTGGAGGG-3’ (synthesized based on the sequence of mmu miR-188-5p; miRBase Accession No. MI0000230); that of miR-SC is 5’- CCUCGUGCCGUUCCAUCAGGUAG-3’; that of miR-124 mimic is 5’-UAAGGCACGCGGUGAAUGCC-3’ (synthesized based on the sequence of mmu miR-124-3p; miRBase Accession No. MI0000828). The antisense 2’-O-methyl oligonucleotide for miR-188 (2’-O-Me-188-AS) was obtained from Integrated DNA Technologies (IDT, CA, USA) or Genolution Pharmaceuticals (Seoul, South Korea). The sequence of 2’-O-Me-188-AS is 5’-rGrCrUrCrGrCrCrCrUrCrCrArCrCrArUrGrCmAmAmGmGmGmAmUmGrUrGrArGrA-3’ (r, RNA base; m, 2’-O-methyl base).

**Whole-cell patch clamp recordings**

For AMPAR-mediated mEPSCs, the vector constructs and microRNA mimics were co-transfected into primary cultured hippocampal neurons (DIV 10-12) using a CalPhos Mammalian Transfection Kit (Clontech Laboratories, CA, USA). miR-124 is one of the most abundant microRNAs expressed in the murine brain54. In previous experiments, miR-124 was used as a negative control for miR-188 in the analyses of mEPSCs. Neurons (DIV 17 - 19) were placed in a recording chamber and continuously superfused (1.5 ml/min) with bath solution (aCSF) containing 127 mM NaCl, 5 mM KCl, 2 mM MgCl2, 2 mM CaCl2, 12 mM glucose, 10 mM HEPES, and 0.001 mM tetrodotoxin at pH 7.3 - 7.4 and 300-305 mOsm as described previously. The NMDA receptor antagonist D-aminophosphonovalerate (20 μM) and GABAA receptor antagonist picrotoxin (100 μM) were added to the aCSF. Whole cell voltage-clamp was performed with a MultiClamp 700B amplifier (Molecular Devices, CA, USA). The recording electrodes (8 - 10 MΩ) were filled with a solution containing 130 mM CsMeSO4, 8 mM NaCl, 0.5 mM EGTA, 10 mM HEPES, 2 mM MgATP, 10 mM phosphocreatine, 5 mM QX-314 and 0.1 mM NaGTP (adjusted to pH 7.2 with CsOH).

**Field EPSP (fEPSP) recordings**

Transverse hippocampal slices (400-μm-thick) were prepared in ice-cold dissection buffer (in mM: Sucrose 213; NaHCO3 26; KCl 2.5; NaH2PO4 1.25; D-glucose 10; Na-pyruvate 2; Na-ascorbate 1.3; MgCl2 3.5 and CaCl2 0.5 bubbled with 95% O2/5% CO2) using a Vibratome (VT1200s, Leica, Germany). The slices were allowed to recover at 36 °C for 1 h in normal artificial cerebrospinal fluid (aCSF) (in mM: NaCl 125; NaHCO3 26; KCl 2.5; NaH2PO4 1.25; D-glucose 10; MgCl2 1.3 and CaCl2 2.5) and thereafter maintained at room temperature. fEPSP recordings were performed by investigator blind to genotype using a MultiClamp 700B amplifier (Molecular Devices, CA, USA) and a Digidata 1440A interface (Molecular Devices, CA, USA). Signals were filtered at 2.8 kHz and digitized at 10 kHz. Resistances of recording pipettes were 2 - 3 MΩ when filled with aCSF. Synaptic responses were evoked at 0.05 Hz with aCSF-filled glass pipettes (0.3-0.5 MΩ) placed in the stratum radiatum and stimulation intensity was adjusted to yield ~40% of a maximal synaptic response. LTP was induced by four episodes (0.1 Hz) of theta burst stimulations (TBS). TBS consisted of ten trains (5 Hz) with each train consisting of four pulses (100 Hz). Data were analyzed using Clampfit software (Molecular Devices, CA, USA) and custom macros written for Igor (WaveMetrics).

**Contextual Fear Conditioning Test**

Contextual fear conditioning was tested as described previously. Each scrambler was connected to an electronic constant-current shock source that was controlled via an interface connected to a Windows 7 computer running EthoVision XT 8 software (Noldus Information Technology, VA, USA). A digital camera was mounted on the steel ceiling of each chamber, and video signals were sent to the same computer for analysis. During training, mice were placed in the conditioning chamber (13 × 13 × 25 cm) for 3 min (for pre-shock) and then received three repetitions of a foot-shock (0.7 mA, 2 sec) at 1 min inter-trial intervals after habituation for 10 min in the same chamber 1 day before the training. On the next day, conditioned mice were placed in the same chamber, and the “freezing” time was measured over periods of 5 min. Conditioned freezing was defined as immobility except for respiratory movements. The total freezing time in the test period was represented as a percentage.

**T-maze Test**

Spontaneous alternation performance was tested using a T-maze, as described previously[^3^](#_ENREF_3) (length of start and goal stems - 30 cm, width - 15 cm, height - 7 cm and 7 × 7 cm center piece). A trial consisted of 2 runs, with a time interval of 2 min. After the mice had been placed in the start arm, the animal was free to choose between both goal arms. As soon as the animal entered one goal arm, the goal arm was closed from the central partition, and the animal was confined to the chosen arm for 30 sec. The animal was then returned to its home cage. After thoroughly cleaning the T-maze with 70 % ethanol, the mice were placed back in the start arm and were free to choose one of the goal arms. The main measure of the test was the alternation ratio, which was defined as the proportion of trials in which alternation occurred (first to the left arm and then to the right arm or vice versa), divided by the total number of trials. A total of 4 trials were conducted over 2 days (2 trials per day).

**Supplemental Experimental Procedures References**

1 Kimura, R. & Ohno, M. Impairments in remote memory stabilization precede hippocampal synaptic and cognitive failures in 5XFAD Alzheimer mouse model. *Neurobiol. Dis* **33**, 229-235 (2009).

2 Kimura, R., Devi, L. & Ohno, M. Partial reduction of BACE1 improves synaptic plasticity, recent and remote memories in Alzheimer's disease transgenic mice. *J. Neurochem.* **113**, 248-261 (2010).

3 Deacon, R. M. J., Nicholas, J. & Rawlins, P. T-maze alternation in the rodent. *Nat. Protoc.* **1**, 7-12 (2006).
